# Experimental Study on the Time-Dependent Characteristics of MLPS Transparent Soil Strength

**DOI:** 10.3390/ma15144990

**Published:** 2022-07-18

**Authors:** Xinzhe Que, Zhao Jin, Yixuan Hou, Yongchao Zhou, Yiping Zhang

**Affiliations:** College of Civil Engineering and Architecture, Zhejiang University, 866 Yuhangtang Rd., Hangzhou 310058, China; quexinzhe@zju.edu.cn (X.Q.); jinzhao@zju.edu.cn (Z.J.); 11812052@zju.edu.cn (Y.H.); zhoutang@zju.edu.cn (Y.Z.)

**Keywords:** transparent soil, strength properties, gas injection test, cracking pressure, influencing factors

## Abstract

The time-dependent characteristics of transparent soil strength, composed of magnesium lithium phyllosilicate, is important for applying a thixotropic clay surrogate. The gas injection method was employed to obtain the strength, represented as cracking pressure, which was then correlated to variables including rest time, disturbance time, and recovery time. Three concentrations (3, 4, and 5%) were tested. The results show that the strength was directly proportional to the rest time, recovery time, and concentration while the disturbance time reversed. The calculated limit strengths for 3%, 4%, and 5% transparent soils were 3.831 kPa, 8.849 kPa, and 12.048 kPa, respectively. Experimental data also showed that the residual strength for higher concentration transparent soil was more significant than the lower ones. The elastic property immediately generated partial strength recovery after disturbance, while the viscosity property resulted in a slow recovery stage similar to the rest stage. The strength recovery rate was also sensitive to concentration. Furthermore, the strength with 3%, 4%, and 5% concentrations could regain limit values after sufficient recovery, which were calculated as 4.303 kPa, 8.255 kPa, and 14.884 kPa, respectively.

## 1. Introduction

Due to their transparency and clay-like features, transparent soils have been considered advanced substitutions of earth soils in numerous geotechnical tests [1,2]. Different transparent materials produce significant discrepancies in physical and mechanical properties [3]. Soft clay could be simulated by researchers utilizing transparent soils [4,5], among which the magnesium lithium phyllosilicate (MLPS), commercially called Laponite RD^®^, was regarded as one of the most suitable materials [6,7]. MLPS transparent soil has numerous applications in simulating the complex rheological properties of soft clay. For example, the scope of deformation and collapse above a tunnel were thoroughly observed in MLPS transparent soil [8]. The development of dendrite cracks, previously invisible, was also observable with MLPS transparent soil [9]. Furthermore, the geometric evolution of fracture surfaces during in situ undrained shear strength tests was improved by MLPS transparent soil [10]. Additionally, Ads et al. studied the projectile penetration as well as the tunnel settlement in MLPS transparent soils due to their internal transparency [11,12].

MLPS transparent soil is a synthetic layered silicate clay mineral available as a white powder, and the chemical formula is written as Na_0.7_Si_8_Mg_5.5_Li_0.3_O_20_(OH)_4_ [13]. Each MLPS particle generally looks like a disk-shaped platelet with a diameter of 25–30 nm and a thickness of around 1 nm [14]. The refractive index of hydrated MLPS was 1.336, nearly equivalent to that of water (1.333), resulting in the magical visibility [15,16]. The fluid-like and solid-like performances are adjustable by controlling the different proportions of MLPS powder [17,18,19]. For high MLPS concentrations, hydration reactions facilitate the strong attraction between the negatively charged faces and positively charged sides of the disks, defined as a “House of cards” [18]. There is ample evidence that MLPS transparent soil’s permeability coefficient, apparent viscosity, and strength characteristics are analogous to soft clay [20,21]. 

The properties of MLPS transparent soil depended closely on the competition between structural construction and deconstruction [18]. The former of which is associated with aging time, including rest time and recovery time, while the latter occurs during an external disturbance. These hydration reactions in MLPS suspensions not only lead to time-dependent changes in viscosity and modulus [22], but also contribute to the transitional behavior from a fluid-like to solid-like state [23]. Low concentrations of MLPS were verified to be in accordance with Newtonian fluid rheology [24]. Therefore, Therefore, concentration and aging were important parameters for MLPS strength [25]. Arachchige et al. utilized simple linear equations to analyze strength with time and concentration by rheological methods, ignoring the complex rheological properties of transparent soils [26]. Bertelsen et al. applied MLPS–water mixtures to study the transition criteria of magma [27]. Biswas et al. studied the damage process by falling spherical balls of different sizes into MLPS and contributed rapid structural changes to applied forces [28]. The thixotropic performance and structure recovery of MLPS–water suspensions were also taken into consideration and were enhanced by concentration changes. Previous work only considered concentrations lower than 4% and recovered relatively short aging times—within seconds to hours [29]. Since the rheological characteristics of MLPS–water suspensions are extensively used, mechanical property variations with aging and thixotropy need further research. However, related research on the rheological properties of MLPS transparent soil strength is still insufficient, especially in how the destruction process affects the long-term recovery process. 

Therefore, one motivation of this paper was to replenish the considerations in MLPS thixotropy and recovery based on the time-depending MLPS strength properties. However, several current techniques available to measure the mechanical properties are difficult to implement. For example, shear rheometry needs expensive machines and pre-training [21]. Meanwhile, the razorblade-initiated fracture test focuses on only the material surface rheology [30] and microbead rheology is only suitable for soft materials [31]. Recently, Zimberlin et al. developed an advanced gas injection method to determine modulus within an arbitrary soft material by pressurizing a single bubble at the needle tip [32]. Interestingly, the pressure obtained from the gas injection method is directly related to material rheology. Therefore, we used this method because of the simplicity in quantifying the material properties. 

The work reported here concerns the time-dependent MLPS strength properties, motivated by the consideration of destruction and reconstruction influence on MLPS strength. Tests were carried out to measure the strength characteristics, considering either multiple aging times (rest time and recovery time) or various disturbance degrees. The relationship between time, disturbance degree, and strength was also studied. The results will improve MLPS as an alternative material for experimental research in geotechnical engineering.

## 2. Experimental Material and Conditions

### 2.1. Material and Apparatus

The concentration of MLPS transparent soil is defined as:

(1) $$C\%=\frac{m_{p}}{m_{p}+m_{w}}\times 100\%$$

where *m_p_* represents the mass of MLPS powder and *m_w_* represents the mass of deionized water.

**Figure 1:**
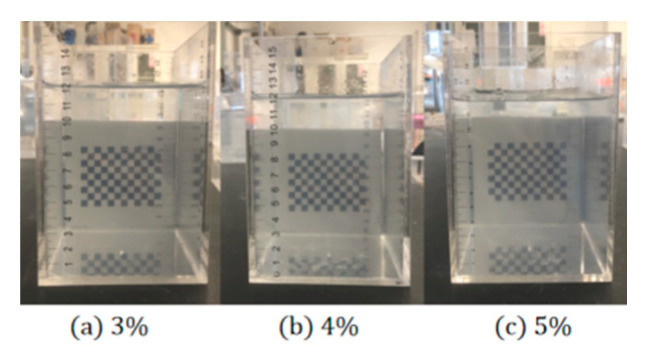
Samples of MLPS transparent soil.

The well-proportioned MLPS powder was vigorously intermixed with deionized water by a stirring cup and then placed into a vacuum tank to remove bubbles. Subsequently, the solution was non-interferential stored in an acrylic box with an insulated layer to maintain an undisturbed state, the height of which was precisely controlled at 12 cm. Since the rheology of MLPS with concentrations lower than 3% behaved quite differently than natural soft soils [27], which were uninteresting and, thus, excluded. Moreover, the indispensable condensation rate for concentrations higher than 5% contributed greatly to non-uniform dispersion. Therefore, the range of concentrations was set at 3%, 4%, and 5%, which was analogous to previous studies [33], as shown in Figure 1.

**Figure 2:**
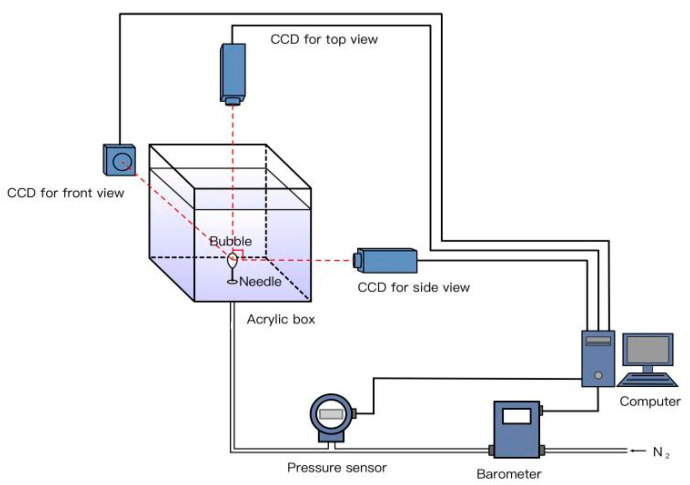
Schematic diagram of the device.

The schematic diagram of the gas injection device is shown in Figure 2. The device included an organic acrylic box whose cross-section was 10 cm × 10 cm; a steel cylinder in which the injected nitrogen gas was initially stored; a pressure sensor (MEACON MIK-P300, Hangzhou, China) located near the 0.26 mm size needle to capture instantaneous pressure fluctuation; a barometer (Sevenstar^®^ CS200A, Beijing, China) was arranged to control the gas flow rate in the whole system at a suitable measuring range of 5 sccm; and a computer, all of which were interconnected by rubber pipes. Three digital cameras (Baumer VCXG-13M, Frauenfeld, Switzerland), all connected to a computer, were utilized simultaneously for the front, side, and top views. Each camera took 90 frames per second and had a matrix of 1024 pixels × 1280 pixels [34].

**Figure 3:**
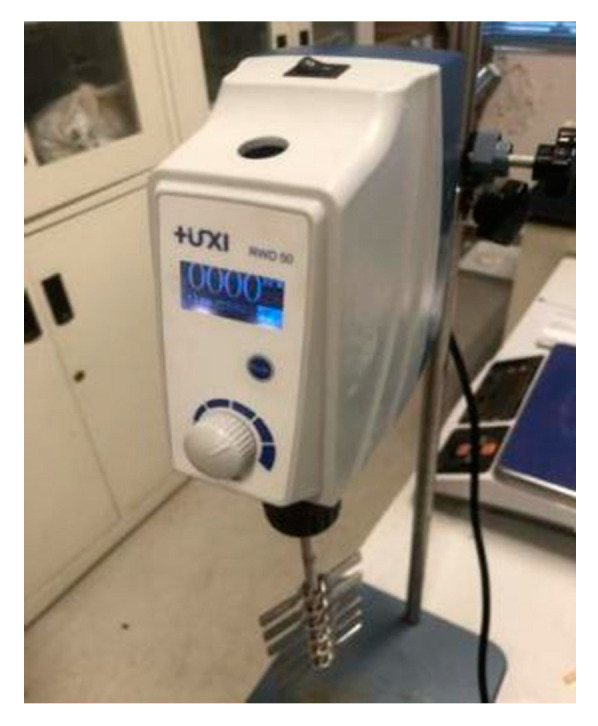
Blender and stirring rod.

The sample prepared for disturbance tests was severely destructed by a stainless steel stirring rod 400 mm long and 8 mm in diameter. The end of the rod had six parallel impellers of 80 mm in diameter and 20 mm in distance that were specially configured by a Huxi RWD50 blender (Figure 3) with 2000 rpm rotational speed to ensure the thorough damage of the sample structure.

### 2.2. Testing Conditions and Procedure

Three concentrations (3%, 4%, and 5%) of MLPS transparent soils were considered in this paper. For each concentration, samples were prepared and divided into three test groups. In order to control the variables, each group only considered one influence. Therefore, several critical values should be defined beforehand. The first definition set 3 days of rest time as a basic condition at which we assumed samples were stable since the experimental results showed that the MLPS samples were in a relatively stable stage at around 3 days, which was consistent with previous studies [35]. Based on that, we used samples with 3 days of rest to conduct disturbance tests. The second definition was setting 60 minutes of disturbance time as a critical value at which we assumed samples were fully destroyed. According to experimental results, at 60 minutes, samples reached a minimum pressure, commonly called the residual pressure (discussed in Section 3.3). Therefore, 60 minutes was regarded as a proper value for a sample to be fully destroyed. Detailed test procedures were:

(1) Rest tests: MLPS samples were respectively rested for 0, 1, 2, 3, 5, 7, and 9 days before getting ready for gas injection tests.
(2) Disturbance tests: at 3 days of rest, every fresh MLPS sample imposed a disturbance of 5, 15, 30, and 60 min separately. The appropriate rotational speed was set at 2000 rpm. After disturbance, the gas injection test was carried out.
(3) Recovery tests: at 3 days of rest, fresh MLPS samples were disturbed for 60 min to be entirely destroyed. The appropriate rotational speed was set at 2000 rpm. When the 60-minute disturbance stopped, it was taken as the beginning of the recovery time in the 0–8 days range. After recovery time, the gas injection test was carried out.

The testing conditions are shown in Table 1.

In this paper, we adopted a similar gas injection method proposed by Zimberlin et al. [32] to investigate material strength. The detailed experimental procedure involved unscrewing the valves of the nitrogen bottle, barometer, and pressure sensor; checking the air tightness of the system; and checking needle permeability. Then, the barometer was adjusted at 5 sccm with the automatic start-up system software. At the same instant, the artificially controlled needle was pushed exactly 2 cm into the MLPS transparent soil in a box through a hole at the bottom. Afterward, the valves of the barometer were closed at an appropriate time of bubble growth. 

The disturbance procedure included penetrating the installed stirring rod into the material, turning on the power supply, and turning off the power supply. Several precautions were utilized to reduce the unavoidable generation of small air bubbles during the disturbance process, which contributed to transparency reduction. First, we penetrated the steel stirring rod deep into the material to reduce the contact with air. Second, we slowed the rotational speed before extracting the steel stirring rod, also to avoid spatter. 

### 2.3. Cracking Pressure Mechanics

**Figure 4:**
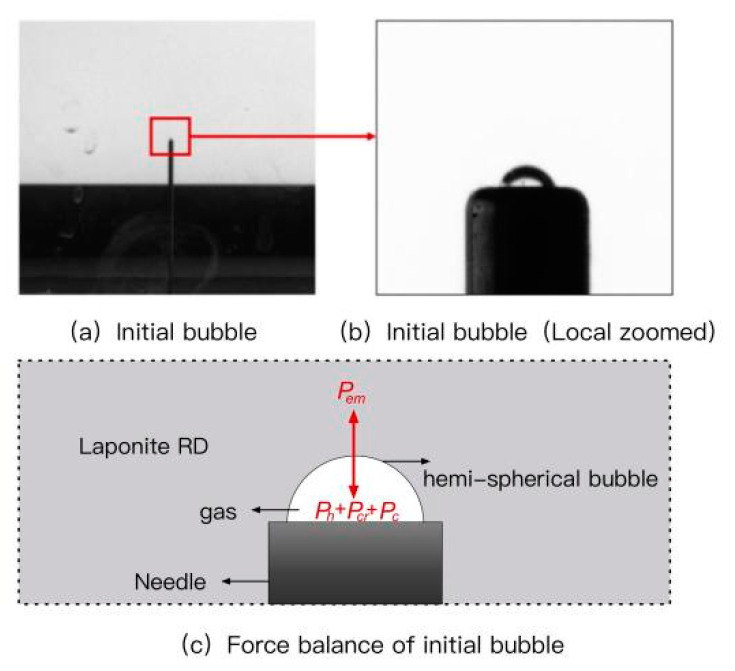
Force balance diagram.

Previous observations showed that the bubble at the needle tip hardly grew until a critical pressure was reached—widely known as cavitation or fracture instability [36,37]. Therefore, the hypothesis was proposed that the initial bubble defect length scale was equivalent to the needle inner radius *r*_0_ [32,38]. This assumption was tested using needles with different inner radii [38]. Furthermore, Zhang et al. used a high-speed camera with 1000 frames per second to capture bubble growth morphology at the needle tip in MLPS transparent soils [39]. As shown in Figure 4c, the initial bubble surface had a semi-spherical shape and was tangent to the needle tip when instability occurred, further proving this assumption.

For the initial growth bubble, the pressure was balanced by the interaction of injection pressure, hydrostatic pressure, capillary tension, and cracking pressure, as shown in Figure 4c. Therefore, the force balance at initial instability was:

(2) $$P_{em}=P_{h}+P_{c}+P_{cr}$$

where *P_em_* represents the maximum pressure at initial instability, *P_h_* represents the hydrostatic pressure, $P_{h}=\rho_{l}gh$, *h* represents the distances from the pinhole to the sample surface, which is constant at 0.10 m. *ρ_l_* represents the sample density, $\rho_{l}=m_{s}/V$. The densities at 3, 4, and 5% were 1.031, 1.042, and 1.053 g/cm^3^, respectively. As discussed before, the bubble’s initial radius *r_0_* was approximately equal to the needle’s inner radius 0.26 mm, σ represents the surface tension whose value we considered constant at 0.0728 N/m. *P_c_* represents the capillary resistance, $P_{c}=2\sigma /r_{0}$, therefore, it is 0.560 kPa. *P_cr_* represents the cracking pressure. Using the neo-Hookean strain energy model, one could relate *P_cr_* to local elastic modulus, $\frac{P_{cr}}{E}=\frac{P_{em}-P_{c}}{E}=\frac{5}{6}-\frac{2}{3\lambda }-\frac{1}{6\lambda^{4}}$, where λ represents the extension ratio of cavity radius and *P_h_* = 0 [32]. The calculated results met satisfaction with the classical cone and plate rheology. Frieberg et al. applied this to summarize the experimental results of critical hydrostatic pressure to the elastic modulus *E* as well as the concentration *C*% [40]. Another important parameter, the critical stress intensity factor, $K_{IC}=P_{cr}\frac{\sqrt{\pi r_{0}}M_{1}}{Q^{*}}$, could also be calculated in MLPS with high concentrations, where *r*_0_ represents the pinhole inner radius, *M_1_* represents a correction factor, and Q* is an approximate expression of the complete elliptic integral of the second kind [39].

The cracking pressure *P_cr_* was used in many previous studies to calculate the rheology of soft materials, such as elastic modulus, and the results were relatively satisfying. However, specific values of mechanical properties were not further considered. We focused merely on strength development. Therefore, the cracking pressure *P_cr_* was adopted in this paper to reflect the strength and prosperity of MLPS transparent soils. 

## 3. Testing Result Analysis

### 3.1. Pressure and Morphology

**Figure 5:**
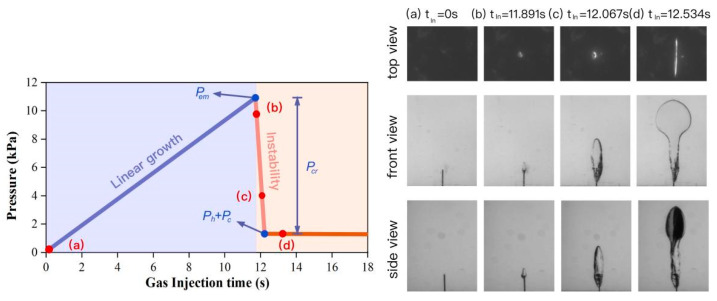
Pressure curve of the gas injection test and corresponding bubble shapes.

An experimental pressure analysis of MLPS transparent soil is exhibited below as an example, which was the 5% concentration rested for 3 days. As shown in Figure 5, the pressure accumulated linearly under the constant gas flow rate until it met a maximum value and then dropped dramatically. During the linear stage, the gas–liquid interface of the target bubble rose along the inner wall of the needle. Since these micro-bubbles were too small to observe by the naked eye, the corresponding photos are similar to Figure 5a. As the accumulated pressure approached the maximum value, a semi-spherical shape gradually formed, as described in Figure 4. At the curve tip, instability happened. Then, the bubble deformed, as shown in Figure 5b–d. The experimental maximum pressure *P_em_* reached 10.942 kPa, and the hydrostatic pressure *P_h_* was calculated as 1.032 kPa. Therefore, the *P_cr_* was calculated as 9.308 kPa. All the other samples shared this similarity.

### 3.2. Influence of Rest Time

**Figure 6:**
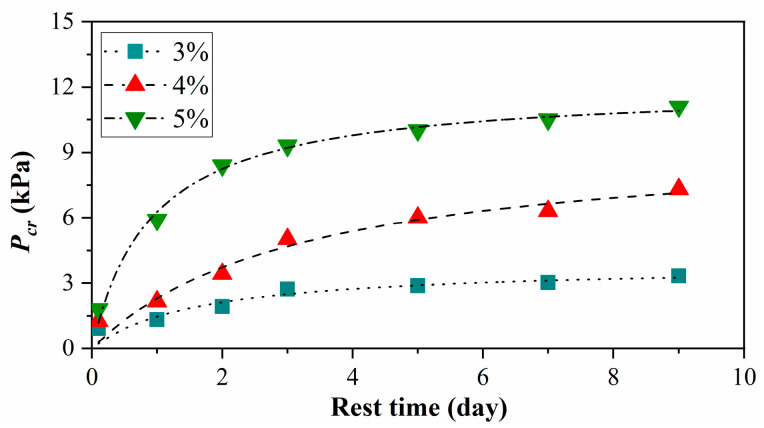
Relationship between cracking pressure and rest time. The cracking pressure *P_cr_* is adopted to reflect the strength prosperity of MLPS transparent soils. The figure shows that the strength increase with the increasing concentrationas well as the rest time. Curve with 5% shows a more strongly increase than curves with 4% and 3%. All curves reach stable stages after enough rest time.

Figure 6 shows the cracking pressure of MLPS transparent soils with different rest times obtained by gas injection tests. The cracking pressure increased with the increasing concentration of transparent soils, indicating that the higher concentration of transparent soils had greater strength at the same rest time. The cracking pressure increased rapidly in the early stage within 2–3 days before reaching a relatively stationary stage at the same concentration. The strength variation of MLPS transparent soil at a lower concentration tended to reach a stable stage faster, especially in 3%, where the strength remained constant after 3 days. Although the early strength variation at higher concentration grew significantly before slowing down, the strength of 4% or 5% still increased continually after 3 days. For unification, the samples in both the disturbance and recovery tests shown in Table 1 rested 3 days before experiments.

The hyperbolic relationship between cracking pressure and rest time *T* in Figure 6 can be written as:

(3) $$P_{cr}^{}=\frac{T}{a+bT}$$

where *a* and *b* are simulation parameters, and 1/*a* represents the physical significance of the initial strength growth rate, the enlargement of which shows a faster strength growth in the initial period, while 1/*b* represents the limit strength (denoted as $P_{cr}^{\infty }$). The two unknown parameters *a*, *b* were obtained by the non-linear least-squares method based on the Levenberg–Marquardt iterative algorithm, which has been widely used in simulations. The same procedure was conducted with other equations below. The fitting lines are shown along with the experimental data (geometric points) in Figure 6. All figures below were expressed in this way for unification. The calculated limit strengths of MLPS transparent soils with 3%, 4%, and 5% were 3.831 kPa, 8.849 kPa, and 12.048 kPa, respectively.

**Figure 7:**
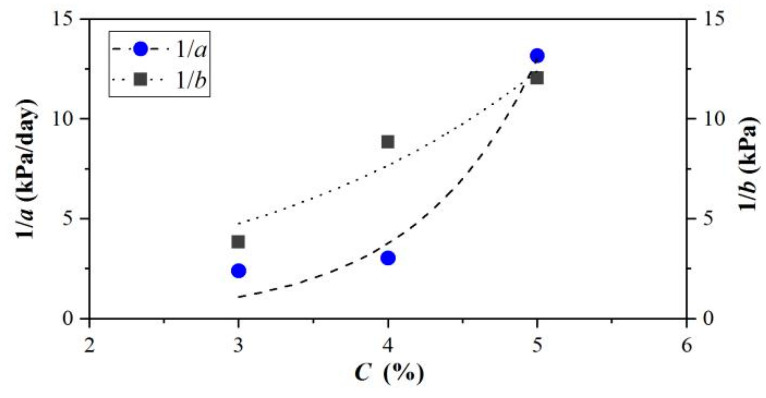
RRelationship between 1/*a*, 1/*b,* and concentration.

The relationship between the parameters *a*, *b,* and the concentration are shown in Figure 7, which can be expressed as:

(4) $$\left\{\begin{array}{l} 1/a=m_{1}e^{n_{1}C}\\ 1/b=m_{2}e^{n_{2}C} \end{array}\right.$$

where *m*_1_, *n*_1_, *m*_2_, and *n*_2_ are simulation parameters obtained from the experimental data. The parameters 1/*a* and 1/*b*, of which the correlation coefficients *R*^2^ were 0.938 and 0.934, respectively, increased with the concentration growth in Figure 7, proving the satisfactory fitting accuracy.

### 3.3. Influence of Disturbance Time

Figure 7 shows that the cracking pressure varies with disturbance time. The MLPS transparent soils rested for 3 days, and the gas injection tests were carried out right after the complement of the disturbance process at 2000 rpm. The cracking pressure decreased with the change in disturbance time, and the strength of transparent soils approached roughly stable at 30 min. The cracking pressure of 3% or 4% dropped gradually to a floor level at 60 min that was nearly equivalent to the initial strength (corresponding to 0 min in Figure 6), indicating the complete destruction of the internal structure. 

Contrary to the phenomenon above, a non-negligible residual strength (denoted as $P_{cr}^{0}$) existed for 5% MLPS transparent soils even under 60 min disturbance. It is commonly known that MLPS particles work as effective multifunctional bond agents and are connected mutually by entangled chains. Therefore, the applied pressure was composed of physical chain pill-out and the disentangled polymer chains [41], and the elastic behavior of strength accounted for changes in polymer chain entropy [42]. The chains were divided into two parts: entanglements and crosslinks; the former were easier to break while the latter were sturdy [42]. At low concentrations, MLPS particles formed entanglements and separated far apart. While at large concentrations, crosslinks dominated and particles formed a sturdy structure, making it difficult to be thoroughly destroyed, and a certain amount of internal structure existed [43]. In this way, the residual strength in high concentrations was much more significant than in low ones.

**Figure 8:**
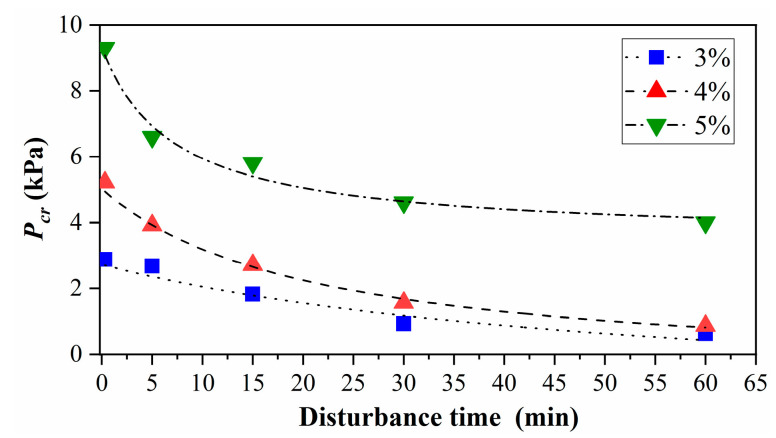
Relationship between cracking pressure *P_cr_* and disturbance time. The *P_cr_* shows decrease since the disturbance process have destroyed part of the the original structure. All curves reach stable stages after enough disturbance time, which are commonly denoted as residual strength. The residual strength in 5% MLPS samples was much more significant than that in 3% and 4%.

The relationship between cracking time and disturbance time *t* (regard the beginning time of disturbance as zero) can be expressed as follows:

(5) $$P_{cr}^{}=-\frac{t}{\alpha +\beta t}+P_{cr}^{}(T_{0})$$

where 1/*α* represents the initial decline rate of the disturbance curve while 1/*β* represents the amplitude of decline, $P_{cr}^{}(T_{0})$ represents the original strength (corresponding to samples with 3-day rest time). As shown in Figure 8, Equation (5) has a good fitting effect, based on which the residual strength is:

(6) $$P_{cr}^{0}=P_{cr}^{}(t\to \infty )=-1/\beta +P_{cr}^{}(T_{0})$$

The residual strengths of 3%, 4%, and 5% transparent soils were −1.695 kPa, −0.630 kPa, and 3.494 kPa, respectively. Strength barely existed with thorough disturbance for low concentrations of MLPS transparent soils (e.g., the residual strengths of 3% and 4% approached 0, which might be caused by the fitting error). In contrast, a considerable residual strength existed after complete disturbance for the high concentration (e.g., 5%), indicating the incomplete disturbance of the internal structure of the high-concentration transparent soil.

**Figure 9:**
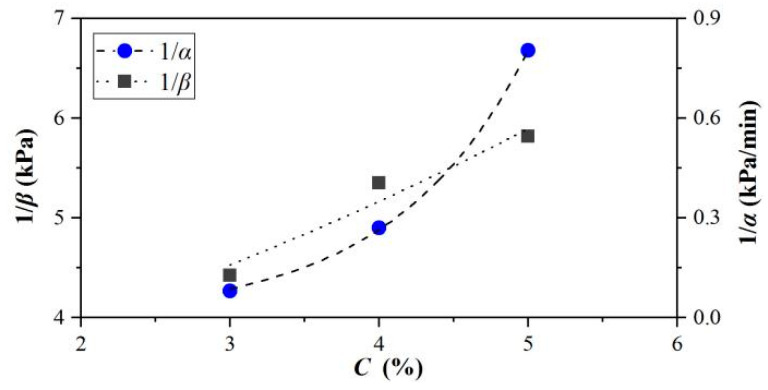
Relationship between 1/*α*, 1/*β*, and concentration.

Parameters *α* and *β* were also functions of concentration, and the relationship can be expressed as:

(7) $$\left\{\begin{array}{l} 1/\alpha =m_{3}e^{n_{3}C}\\ 1/\beta =m_{4}e^{n_{4}C} \end{array}\right.$$

where *m*_3_, *n*_3_, *m*_4_, and *n*_4_ are simulation parameters calculated from the experimental data. Both 1/*α* and 1/*β* increased with the concentration change in Figure 9, where the favorable correlation coefficients *R*^2^ were 0.987 and 0.949, respectively.

### 3.4. Influence of Recovery Time

**Figure 10:**
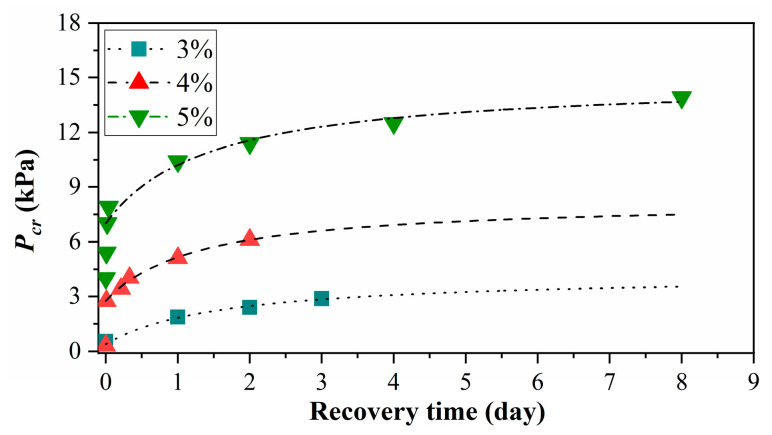
Relationship between cracking pressure *P_cr_* and recovery time. The *P_cr_* values near the origin of horizontal coordinates represent the elastic recovery, which become significant at high concentrations. And then all the curves are similar to the strength growth in the rest period and reach stable stages at the large recovery time.

Figure 10 shows that cracking pressure varies with recovery time, where the MLPS transparent soils had rested for 3 days and were disturbed at 2000 rpm for 60 min. Additional recovery time was considered to testify to thixotropy. As shown in Figure 10, the cracking pressure recovered instantaneously after the completion of disturbance, whose response was homology to the elastic recovery and denoted as $P_{cr}^{e}$ and developed slowly later, whose response was similar to the strong growth in the rest period. The elastic recovery stage was probably related to the residual structure of MLPS materials since the recovery speed was directly proportional to the concentration. Thus, the disentangled MLPS chains were easier to find neighbors in high concentration materials since these MLPS particles had a closer spatial distribution. This phenomenon led to structure rebuild and strength recovery. Therefore, one can frequently find that two recovery stages existed after disturbance. The relationship between the cracking pressure after elastic recovery and recovery time *t′* (regard the ending time of disturbance as zero) can be expressed as:

(8) $$P_{cr}^{}=\frac{{t^{\prime }}_{}}{A+Bt^{\prime }}+P_{cr}^{e}+P_{cr}^{}(T_{1})$$

where 1/*A* represents the strength initial recovery rate while 1/*B* represents the cracking pressure growth with additional recovery time, both related closely with time responding to viscosity response and denoting viscosity recovery pressure. $P_{cr}^{}(T_{1})$ represents the cracking pressure right after the disturbance (regard the strength after 60 min disturbance in experiments).

Figure 10 shows that Equation (8) with experimental data could realize a good fitting effect, where the calculated viscosity recovery pressures (1/*B*) of transparent soil strengths at 3%, 4%, and 5% were 3.773 kPa, 5.494 kPa, and 7.874 kPa, respectively.

Based on Equation (8), one can obtain the corresponding limit cracking pressure (the limit strength):

(9) $$P_{cr}^{\infty }=1/B+P_{cr}^{e}+P_{cr}^{}(T_{1})$$

The elastic recovery pressures of 3%, 4%, and 5% in Figure 10 were taken into consideration on 0.130 kPa, 1.864 kPa, and 3.002 kPa, respectively, and the corresponding limit strengths were 4.303 kPa, 8.255 kPa, and 14.884 kPa, near equivalent to the limit strength calculated by Equation (5). This regularity indicated that the strength of disturbed transparent soils could fully recover the initial strength level without disturbance.

**Figure 11:**
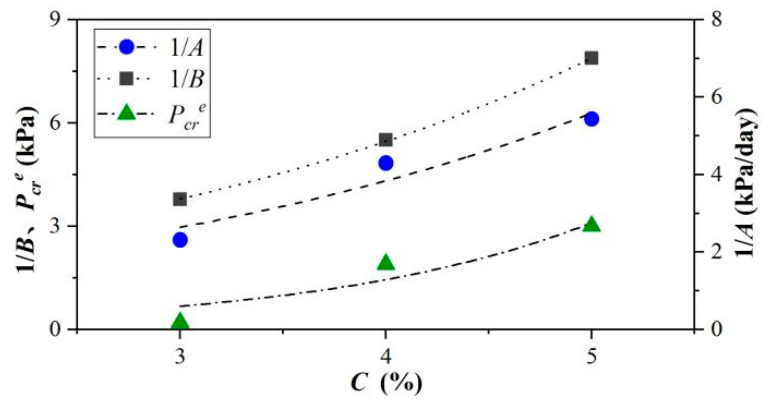
Relationship between 1/*A*, 1/*B*, $P_{cr}^{e}$ and concentration.

The parameters 1/*A*, 1/*B,* and $P_{cr}^{e}$ reflect exponential growth with the concentration in Figure 11, and the relationships can be written as:

(10) $$\left\{\begin{array}{l} 1/A=m_{5}e^{n_{5}C}\\ 1/B=m_{6}e^{n_{6}C}\\ P_{cr}^{e}=m_{7}e^{n_{7}C} \end{array}\right.$$

where *m*_5_, *n*_5_, *m*_6_, *n*_6_, *m*_7_, and *n*_7_ are simulation parameters, and the correlation coefficients of 1/*A*, 1/*B,* and $P_{cr}^{e}$ in Figure 11 were 0.933, 0.999, and 0.891, respectively.

### 3.5. Cracking Pressure Variation

**Figure 12:**
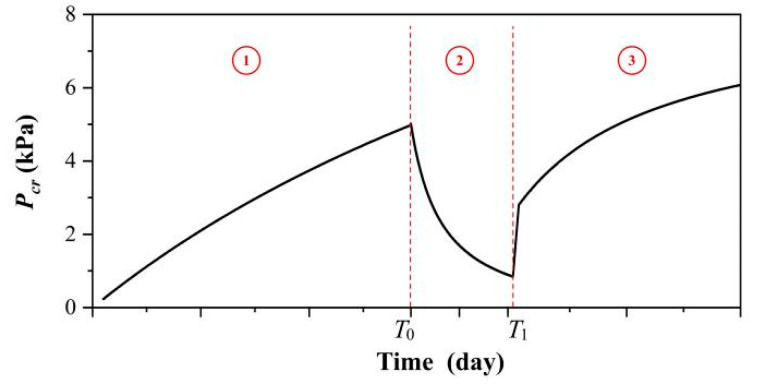
The curve of cracking pressure versus time. The curve could be divided into three parts: ① represents the rest period where the strength grows with time, ② represented the disturbance period where the destroyed structure accounted for strength decrease, and ③ represents the recovery period which corresponds to the structure rebuild and strength growth.

The strength variation during rest, disturbance, and recovery of MLPS transparent soil are combined in Figure 12, where ①, ②, and ③ represent the rest period, disturbance period, and recovery period, respectively. A consolidated equation was used when valuing the strength variation between Equations (3), (5), and (9), which can be expressed as:

(11) $$P_{cr}(T)=\left\{\begin{array}{l} \frac{T}{a+bT},\;(0\leq T\leq T_{0})\\ -\frac{T-T_{0}}{\alpha +\beta (T-T_{0})}+P_{cr}^{}(T_{0}),\;(T_{0}<T\leq T_{1})\\ \frac{T-T_{1}}{A+B(T-T_{1})}+P_{cr}^{e}+P_{cr}^{}(T_{1}),\;(T_{1}<T) \end{array}\right.$$

where *T_0_* represents the disturbance initiation, while *T_1_* represents the disturbance end.

## 4. Conclusions

This study focused on the time-dependent properties of MLPS transparent soil strength, and the objective was to replenish the bare consideration in MLPS thixotropy and recovery. The critical cracking pressure, obtained by the gas injection method, was used to investigate the development of MLPS transparent soil strength. Influencing factors, such as aging time, concentration, and thixotropy, were considered to better serve MLPS as an alternative material for experimental research in geotechnical engineering. The following conclusions can be drawn:

(1) The MLPS strength experiences rapid change and then stabilizes, which is consistent with aging behavior. The strength is proportional to concentration, i.e., MLPS with higher concentrations possess larger strength with equivalent rest time. Moreover, the calculated limit strengths of MLPS transparent soils with 3%, 4%, and 5% were 3.831 kPa, 8.849 kPa, and 12.048 kPa, respectively.
(2) Disturbance contributes greatly to MLPS strength descent. The residual strength for high concentrations was much more significant than for low ones, indicating the different thixotropic reactions caused by structure deconstruction.
(3) The strength recovery for disturbed MLPS with high concentration consists of both elastic and viscous recovery. The former response is probably caused by crosslink re-organization, and the latter accounts for entanglement re-connection. Under enough recovery time, the strengths of 3%, 4%, and 5% MLPS were restored to 4.303 kPa, 8.255 kPa, and 14.884 kPa, respectively, showing the structure recoverability.

## Figures and Tables

**Table 1:** Design of test conditions.

Groups	Concentration (%)	Rest Time (Day)	Disturbance Time (min)	Rotational Speed (rpm)	Recovery Time (Day)
rest tests	3%	0, 1, 2, 3, 5, 7, 9	0	0	0
	4%				
	5%				
Disturbance tests	3%	3	5, 15, 30, 60	2000	0
	4%				
	5%				
Recovery tests	3%	3	60	2000	0, 1, 2, 3
	4%				0, 0.01, 0.21, 0.33, 1, 2
	5%				0, 0.01, 0.02, 0.04, 1, 2, 4, 8

## Data Availability

Not applicable.
